# Enteric Pathogen-Plant Interactions: Molecular Connections Leading to Colonization and Growth and Implications for Food Safety

**DOI:** 10.1264/jsme2.ME13139

**Published:** 2014-05-23

**Authors:** Betsy M. Martínez-Vaz, Ryan C. Fink, Francisco Diez-Gonzalez, Michael J. Sadowsky

**Affiliations:** 1 Department of Biology, Hamline University, St. Paul, MN 55104, USA; 2 Department of Food Science and Nutrition, University of Minnesota, St Paul, MN 55108, USA; 3 Biotechnology Institute, University of Minnesota, St Paul, MN 55108, USA; 4 Department of Soil, Water and Climate, University of Minnesota, St Paul, MN 55108, USA

**Keywords:** foodborne pathogens, transcriptome, fresh produce, lettuce, food safety

## Abstract

Leafy green vegetables have been identified as a source of foodborne illnesses worldwide over the past decade. Human enteric pathogens, such as *Escherichia coli* O157:H7 and *Salmonella*, have been implicated in numerous food poisoning outbreaks associated with the consumption of fresh produce. An understanding of the mechanisms responsible for the establishment of pathogenic bacteria in or on vegetable plants is critical for understanding and ameliorating this problem as well as ensuring the safety of our food supply. While previous studies have described the growth and survival of enteric pathogens in the environment and also the risk factors associated with the contamination of vegetables, the molecular events involved in the colonization of fresh produce by enteric pathogens are just beginning to be elucidated. This review summarizes recent findings on the interactions of several bacterial pathogens with leafy green vegetables. Changes in gene expression linked to the bacterial attachment and colonization of plant structures are discussed in light of their relevance to plant-microbe interactions. We propose a mechanism for the establishment and association of enteric pathogens with plants and discuss potential strategies to address the problem of foodborne illness linked to the consumption of leafy green vegetables.

## Introduction

The contamination of fresh vegetables with enteric pathogens has reached concerning proportions in recent years. Numerous outbreaks associated with the consumption of contaminated lettuce, spinach, and various types of sprouts have been reported in the United States, Canada, Asia, and Europe (Table 1). One of the largest outbreaks of foodborne diseases ever described may have been linked to the consumption of white radish sprouts contaminated with *E. coli* strain O157:H7. Approximately 10,000 cases were reported in at least 14 different locations in Japan; the most affected individuals were children (96). The consumption of contaminated bagged spinach infected more than 200 individuals with *E. coli* O157:H7 in 26 states and one Canadian province in September 2006 (21). A similar multistate outbreak was reported in late 2011, with 58 cases of food poisoning being linked to the presence of *E. coli* O157:H7 in romaine lettuce in five different cities in the United States (84). Fenugreek seeds contaminated with *E. coli* O104:H4 more recently infected over 3,800 individuals in Germany and France (39, 45). These major outbreaks illustrate the risks associated with the consumption of leafy greens as well as the food safety challenges they present for consumers and agricultural producers.

The scientific community has advanced understanding of foodborne bacteria contamination in agricultural and processing environments in the past few years. Initial studies examined bacterial survival in soils, manure, and plants, the ability of harvesting and cutting tools to sequentially contaminate produce, the effectiveness (or lack thereof) of some common sanitizers, and the possibility of utilizing novel technologies, such as irradiation and ozone to enhance the safety of produce (44, 47). In spite of the important findings revealed by these studies, the molecular mechanisms by which foodborne pathogens establish themselves in the plant environment have received the least attention and have yet to be elucidated in detail.

The availability of modern and high throughput molecular tools has provided researchers with the opportunity to study the molecular fabric of microorganisms residing in natural environments and gain an understanding of the intimate interactions between foodborne pathogens and plants. This review summarizes recent findings on the molecular interactions between enteric pathogens and leafy green vegetables. We begin by briefly describing the routes of contamination and factors influencing the survival and persistence of enteric pathogens in fresh vegetables. We then present a synopsis of recent studies that examined the molecular events mediating the interactions of *Salmonella* and *E. coli* O157:H7 with leafy green vegetables. The microbial genes involved in the interactions of foodborne pathogens with fresh produce are discussed along with the global gene expression profiles associated with their persistence and the colonization of plant structures. We propose a mechanism for the establishment of *E. coli* and other enteric pathogens in plants and discuss potential strategies to address the problems of foodborne illnesses linked to the consumption of leafy green vegetables.

## Enteric pathogens in the environment

### Routes of contamination

**Fig. 1:**
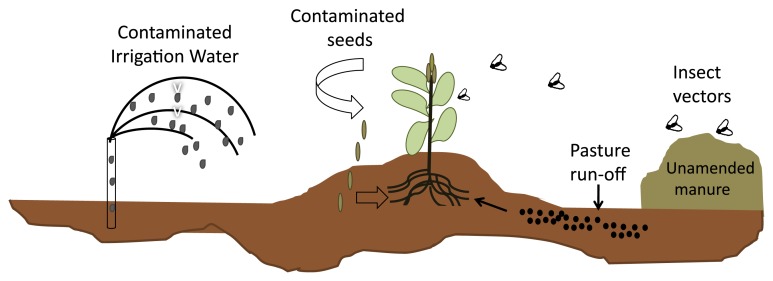
Enteric pathogens in leafy greens: routes of contamination. Representation of the conditions that can cause the contamination of leafy greens with *Enterobacteria* in the pre-harvest environment.

Outbreaks of foodborne diseases due to the consumption of leafy green vegetables have been linked to several sources of contamination, including water, raw or inadequately amended manure, and insect vectors (Fig. 1). Water is an important source of contamination in pre- and post-harvest environments. In the field, runoff from animal pastures and irrigation with contaminated water are considered as primary sources of enteric bacteria in the aerial tissues and root systems of fruit and vegetables. Contaminated water can lead to the internalization of human pathogens into plants when applied to the surface of leafy greens. Mitra *et al.* (68) reported that *E. coli* O157:H7 was internalized in spinach plants sprinkled with contaminated water and survived in the phylloplane for 14 days with an increase in titers and areas of colonization being observed over that time. These effects were not observed when spinach plants were inoculated by soil drench or tissue stabbing methods (68), which indicated that the likelihood of internalizing enteric pathogens increased when these organisms were introduced by water sprinkling systems versus direct application into the soil.

The addition of un-amended manure or raw animal feces as fertilizers to soils increases the likelihood of microbial contamination of crops. Enteric bacteria survive for long periods of time in manure and feces and can, therefore, provide a substantial inoculum for plants growing in agricultural fields. The 2006 bagged-spinach outbreak is an excellent example of this problem. The pathogenic *E. coli* strains isolated from infected patients were identical to O157:H7 strains obtained from cattle and feral swine in a California Salinas Valley ranch near the spinach fields implicated in the outbreak (54). Although the exact mode of spinach contamination was never elucidated, it was speculated that the direct fecal deposition on or indirect contamination of soils and surface waterways by livestock led to the contamination of spinach plants by *E. coli* O157:H7.

Insects are frequently found in manure piles, feedlots, and other habits near fields containing leafy green vegetables. Investigations on the role of filth flies as insect vectors in the contamination of fresh produce revealed that a large number of flies caught near agricultural fields were carriers for *E. coli* O157:H7. Under laboratory conditions, houseflies were shown to transfer pathogenic *E. coli* from agar plates to spinach plants (88). Wasala *et al.* (95) investigated the deposition of *E. coli* O157:H7 by domestic flies onto spinach and demonstrated the multiplication of bacteria in regurgitation spots found on the plant surface. Therefore, it was hypothesized that regurgitation spots may have provided a nutrient source for the survival of pathogenic *E. coli* on spinach leaves. Taken together, these findings suggest that insect vectors, especially houseflies, may be vehicles for the transmission and persistence of foodborne pathogens in leafy green vegetables.

Post-harvest processing also provides sources for the contamination of vegetables by enteric pathogens. Prior to entering the marketplace, fruits and vegetables pass through various pieces of equipment and are subjected to several washing procedures. Recent studies have implicated cutting equipment, shredding, conveying, fluming, and dewatering as sources for the contamination of fresh produce with human pathogens (16, 17, 93). Post-harvesting practices can damage the surfaces of leafy greens. Injured lettuce and spinach have been shown to provide favorable conditions for the growth and dissemination of *E. coli* O157:H7 and *Salmonella enterica*.

Contaminated water is also a concern in the post-harvest processing of fruits and vegetables. Washes with contaminated water were reported to be the cause of a *S. enterica* sv. Newport outbreak in mangoes from a Brazilian farm (83). In addition, a study on the sources of melon rind contamination at processing facilities indicated that water of poor microbial quality was the cause of a large multistate outbreak of *Salmonella enterica* in cantaloupes (43).

### Survival and persistence in soil

The ability of enteric pathogens to colonize leafy green vegetables has been linked to their survival in environments outside the animal host. Numerous studies have investigated the survival and population dynamics of human pathogens in extraintestinal environments, especially soil. Early research by Islam *et al.* (51, 52) reported the survival of *S. enterica* sv. Typhimurium and *E. coli* O157:H7 for over 200 days in soils amended with contaminated compost. Two different studies using manure-amended soils also found that *E. coli* O157:H7 and *S. enterica* survived for 211 and 332 days, respectively (41, 101). In contrast, other studies reported shorter survival times for these pathogens in soil (8, 73). These discrepancies have been attributed to different experimental conditions, inoculation methods, and microbial quantification strategies. In addition, the survival of enteric bacteria in soils may be affected by various factors, including soil edaphic properties, weather, interactions with other microbes, and physiological differences amongst bacterial strains. For example, Franz *et al.* (41) found that survival in soil varied among different strains of *E. coli* O157:H7 and was dependent on the origin of the bacterium and their oxidative capabilities. Human isolates of O157:H7 survived longer than their animal counterparts. In addition, strains that survived for longer periods oxidized propionic acid, α-ketobutyric acid, and α-hydroxybutyric acid at higher rates than short-lasting strains.

Environmental conditions play a critical role in the survival of enteric pathogens. A study by Strawn *et al.* (87) described several external factors that affected the frequency of isolation and prevalence of enteric pathogens in fruit and vegetable farms. Soil topography, moisture, and proximity to water sources increased the chances of isolating enteric bacteria from vegetable farms. This was consistent with the findings of a previous study, which demonstrated that *Salmonella* and *E. coli* O157:H7 persisted longer in soil with a high moisture level than in dry soil (24, 48, 72).

Protozoa have also been shown to play an important role in the survival of *Salmonella* in soils. Brandl *et al.* (13) examined the interaction of *S. enterica* sv. Thompson with the soil-borne protozoa *Tetrahymena* and observed a non-destructive interaction between these organisms. *Salmonella* sv. Thompson was phagocytized by *Tetrahymena*, but evaded digestion and remained viable in the food vacuoles of this organism. The protozoan encapsulation of *Salmonella* may provide a protective effect that prolongs the survival of this bacterium in contaminated environments such as manure-amended soils (14). This proposition is in accordance with previous studies, which reported a decrease in the levels of *S. Typhimurium*, and this correlated with the growth of protozoa in soils (44). The presence of *Salmonella* within intracellular protozoan vacuoles may contribute to its survival in soil and also to the underestimation of the actual numbers of this pathogen in contaminated environments.

In summary, although many environmental factors can impact on the persistence of enteric bacteria in the environment, *Salmonella* and EHEC can survive long enough to contaminate plant roots, leaves, and fruit and gain access to the human host.

### Occurrence and persistence in fresh produce

Survey studies designed to investigate the presence of enteric pathogens in fresh produce have shown that contamination with *E. coli* and *Salmonella* occurs infrequently and at low levels (7, 58, 69, 70). The contamination of leafy greens with human pathogens has recently been associated with the long-term survival of these bacteria in seeds. Van der Linden *et al.* (92) reported that both *E. coli* O157:H7 and *S. enterica* were recovered from stored butterhead lettuce seeds two years after the initial inoculation. The germination of stored contaminated seeds yielded seedlings that tested positive for the presence of *E. coli* and *Salmonella*. These findings indicated that both pathogens have the ability to persist in seeds and proliferate on the seedlings. Therefore, infected seeds and seedlings should be considered as a source of contamination and a factor that facilitates the persistence of enteric bacteria in the vegetable environment.

Delaquis *et al.* (27) reviewed a range of field, greenhouse, and laboratory studies that examined the fate and survival of *E. coli* O157:H7 on growing lettuce and spinach plants (roots and leaves) and also commercially grown leafy green vegetables. Since the experimental objectives, design, bacterial strains, and analytical methods used in these studies were so diverse, it confounded a complete understanding of the factors controlling the fate of *E. coli* in these environments. The frequent association of *E. coli* and *Salmonella* with worldwide food poisoning outbreaks strongly suggests that these microbes possess mechanisms for long-term survival and growth in both the phyllosphere and rhizosphere of leafy green vegetables (1, 51, 86). The findings of recent studies indicated that the persistence of enteric pathogens in or on vegetables was influenced by many factors including the endophytic colonization of the plant host and induction of a viable, but non-culturable (VNBC) state upon association with the phyllosphere (29, 34, 99).

Early studies described the internalization of *E. coli* and *Salmonella* in lettuce and spinach plants following the cultivation of seeds contaminated with these organisms (53, 94). Franz *et al.* (40) also reported the internalization of *E. coli* O157:H7 and *Salmonella* in lettuce plants grown in contaminated soils and hydroponic systems. These findings indicated that the internalization of human pathogens in leafy green vegetables can occur through different sources of contamination and may involve diverse plant entry mechanisms. Internalization has been proposed to occur by two different routes: uptake through the root system along with water (apoplastic entry) or entry in natural openings on the plant surface (including stomata and lenticels), or sites of biological or physical damage. The apoplast refers to the free diffusional space outside the plasma membrane that facilitates water movement inside plants. Once internalized, human pathogens may employ several mechanisms to exploit plant nutrients and ensure their persistence inside tissues. One hypothesis suggests that enteric bacteria living in the apoplastic space of the plant are able to degrade cell wall polysaccharides to obtain sugars and other essential compounds (26). Another possibility is that the survival of internalized enterics is enhanced by the presence of natural endophytes that can degrade cell wall components and plant nutrients that provide carbon and energy sources (26). The mechanisms mediating the internalization of human pathogens in leafy greens have yet to be elucidated in detail. Nevertheless, a recent study suggests that the preferential localization of *E. coli* O157:H7 near the stomata is mediated by the same type three secretion system (TTSS) used by this pathogen to colonize the gut epithelium (82). In contrast, the localization of *Salmonella* near the stomata was shown to be dependent on the bacterial chemotactic responses in and illumination of leaf tissues (59).

While several studies suggested that internalization may be a common consequence of the contamination of leafy greens with enteric pathogens, others indicated that this phenomenon does not occur frequently and depends on several conditions (33, 35, 59, 68, 99, 102). Zhang *et al.* (102) recently used stringent surface-disinfection methods, three types of lettuce, five *E. coli* strains, and different types of inoculation to study the factors that contribute to the internalization of enteric pathogens in vegetables. The study found a complete lack of internalization under all the experimental conditions examined. Erickson *et al.* (33) also showed that the internalization of *E. coli* O157:H7 in field-grown leafy greens was uncommon at the population levels of bacteria normally found in soils. More recently, Wright *et al.* (99) reported the extent of internalization of *E. coli* strains O157:H7 and MG1655 in lettuce and spinach plants. This study showed that *E. coli* O157:H7 cells were easily internalized in lettuce and spinach, and colonized the apoplast. In comparison, only a few cells of *E. coli* MG1655 were recovered from these plants and no internalized bacteria were evident with this strain (99). These findings suggest that the internalization of enteric pathogens is a complex phenomenon that may be limited to specific bacterial strains. The occurrence and extent of internalization may be influenced by environmental and genetic factors as well as experimental conditions.

Enteric bacteria can enter a state of dormancy upon exposure to harsh environments; this condition is referred to as the viable but non-culturable state (VBNC). Bacteria can remain in the VBNC state for long periods of time, during which they switch off most activities characteristic of growing organisms, but maintain a low level of metabolic activity without being capable of growing on typical microbial media (74). Several studies have investigated the ability of foodborne pathogens to enter the VBNC state in different food matrices and under conditions mimicking storage and processing in the food production environment. Makino *et al.* (65) reported that enterohemorrhagic *E. coli* could enter the VBNC state when interacted with salted salmon roe. In addition, *Salmonella spp.* and *Listeria monocytogenes* have been shown to evolve towards the VBNC state under certain conditions associated with the food production chain (31). *Listeria* cells became VBNC when associated with parsley leaves under dry conditions (31). Dinu *et al.* (29) recently reported that the VBNC state in *E. coli* O157:H7 was induced in the phyllosphere of lettuce upon exposure to low temperatures. These findings suggest that human pathogens use the viable non-culturable state as a strategy to survive and persist in leafy greens. This phenomenon presents an alarming food safety concern because entry into the VBNC may lead to the underestimation of pathogenic bacteria in the surfaces of vegetables and prevent the detection of contaminated fresh produce.

## Factors influencing plant colonization by foodborne pathogens

One of the most important questions regarding the persistence of human pathogens on leafy greens is whether genetic traits and/or environmental conditions make plants more vulnerable to colonization by these bacteria. Some of the factors that have been proposed to influence the colonization of vegetables by enteric pathogens include plant and bacterial genotypes, the physiological state of the plant (including leaf age and surface damage), and interactions with resident bacteria in the phyllosphere. A study by Brandl *et al.* (12) demonstrated that enteric pathogens could multiply on lettuce leaves and that bacterial population sizes were strongly dependent on leaf age. *E. coli* O157:H7 and *Salmonella* populations were shown to be consistently larger in young lettuce leaves than in middle leaves harvested from mature lettuce heads (12). These findings indicate that leaf age is an important factor in the development of bacterial communities on vegetable surfaces, and also that younger leaves are at greater risk of contamination with human pathogens.

### Plant genotype effects

Several studies have examined the effects of plant cultivars on the metabolic activity and colonization of plants by potential foodborne pathogens. Quilliam *et al.* (79) investigated the metabolic activity of *E. coli* strain O157:H7 in the rhizosphere and phyllosphere of 12 different lettuce cultivars. The metabolic activity levels of this pathogen in different types of lettuce have been proposed to influence its colonization potential in agricultural environments. Among the lettuce cultivars examined, *E. coli* O157:H7 had the highest and lowest levels of metabolic activity in “Vaila” and “Dazzle” cultivars, respectively. The effects of the cultivar on metabolic activity were observed in seedlings grown in a gnotobiotic environment (*in vitro*) as well as in the rhizosphere of plants grown in compost. The *E. coli* O157:H7 cells internalized or tightly attached to lettuce leaves had higher metabolic activity than the cells weakly bound to the leaf surface. Based on these findings, it was proposed that pathogenic *E. coli* cells not only persisted on the phyllosphere and rhizosphere of lettuce, but also actively metabolized plant-derived nutrients (79). The metabolic activity of pathogenic *E. coli* in the rhizosphere of different lettuce cultivars was undetectable upon removal of the host plant; the re-sowing of seedlings led to the recovery of metabolic activity and successful colonization of new plants. Based on these findings, plant compounds such as root exudates, root architecture, and microbial communities may play an important role in mediating the effects of cultivars on the metabolic activity of pathogenic *E. coli* (79).

A similar study by Mitra *et al.* (68) reported the effects of cultivars on spinach plants infected with *E. coli* O157:H7. Colonization by this pathogen was compared among three spinach varieties with differing leaf morphologies; these included the rough-surface cv. Tyee, semisolid-leaf cv. Space, and cordate-leaf cv. Bordeaux. Cultivar Tyee showed the greatest level of bacterial colonization, while Bordeaux had the lowest bacteria titers of all cultivars. Discrepancies in bacterial cell numbers were attributed to differences in leaf topology (68). The surfaces of Tyee’s leaves had more prominent ridges and valleys than those of Space and Bordeaux. These features may create protective niches that favor bacterial survival in and colonization of the leaf surface. Alternatively, the effects of cultivars in spinach plants could be due to differences in the nutrient composition of the phylloplanes on the spinach varieties studied, and it is possible that the nutrients present in Tyee’s leaves may be more suitable for the growth of *E. coli* O157:H7(68).

The colonization of tomato plants by *Salmonella* has been shown to be cultivar-dependent and variable amongst species (5). In contrast, a study on the interactions of this bacterium with different lettuce varieties revealed the absence of a correlation between plant genotypes and microbial colonization levels (57). In spite of the lack of significant cultivar effects, *S. enterica* colonization was negatively correlated with the species richness of the microbial communities present in the lettuce varieties examined. This finding was consistent with the proposed role of phytobacteria as an important factor in the association between and establishment of human pathogens in leafy green vegetables.

### Interactions with resident phytobacteria

The colonization and establishment of human enteric pathogens in fresh vegetables involves complex interactions with the microbial flora of the host plant. Resident phytobacteria can be beneficial or detrimental to the survival of pathogens on or in leafy greens. Cooley *et al.* (25) described the effects of two epiphytes isolated from *Arabidopsis*, *Wausteria paucula* and *Enterobacter asburiae*, on the survival and growth of O157:H7 in lettuce. The presence of these epiphytes had opposite effects on lettuce-associated *E. coli* populations. *W. paucula* enhanced the survival of the pathogen 6-fold, while *Ent. asburiae* decreased it by 20- to 30-fold. The inhibitory effects observed in the *Ent. asburiae*-O157:H7 interaction were thought to be due to competition between these microorganisms for the nutrients available in the host plant. This hypothesis was supported by the finding that *E. coli* O157:H7 and *Ent. asburiae* mostly utilized the same carbon sources, amino acids, and organic acids present in the lettuce exudates (25). The inhibition of *E. coli* O157:H7 growth was observed in the absence of *Ent. asburiae*, using pre-conditioned plant exudates previously exposed to this phytobacterium. Hence, it is possible that this organism may produce a diffusible factor to outcompete pathogenic *E. coli* in response to nutrient limitations in the plant environment.

The enhanced survival of *E. coli* O157:H7 in the presence of *W. paucula* was observed in the foliage of lettuce, but was absent in plant exudates and the rhizosphere. Unlike *Ent. asburiae*, the carbon sources utilized by *W. paucula* were very different from those preferred by *E. coli* O157:H7. Therefore, commensalism has been proposed for *W. paucula* and pathogenic *E. coli*. The mechanisms of this interaction have yet to be understood; however, it is tempting to speculate that *W. paucula* assists in the establishment of *E. coli* in lettuce by modifying plant nutrients or by creating a polysaccharide matrix that facilitates the attachment of cells to the leaves and roots (25).

Leaf surfaces present a hostile environment to immigrant bacteria due to lack of moisture and limited nutrients, whereas modifications to the plant surfaces by indigenous bacteria may facilitate the establishment and survival of enteric pathogens in this environment. *Salmonella* strains have been shown to have lower epiphytic fitness in the phyllosphere than other human pathogens (10, 15). This is also true for other environments. A recent study by Poza-Carrión (78) showed that *S. enterica* sv. Montevideo had higher survival rates on leaves inoculated with indigenous bacteria than on plants with no prior exposure to these epiphytes. This findings indicated that the presence of indigenous bacteria on leaf surfaces was a relevant factor for the survival of invading enteric bacteria in the phyllosphere. This protective effect was dependent on the co-localization of *Salmonella* cells within bacterial aggregates present on the leaf surface. The enhanced survival of *Salmonella* in the presence of indigenous epiphytes has also been observed in lettuce and cilantro leaves. The mechanisms underlying the beneficial interactions of *Salmonella* with leaf epiphytes have yet to be elucidated. Modifications to the leaf microenvironment by resident bacteria remain the most common explanation for this phenomenon. The formation of protective bacterial aggregates on leaf surfaces is also a possible mechanism for the survival of bacteria in the plant environment. Such aggregates may help cells to cope with nutrient limitations and other challenges commonly posed by the phyllosphere (78). In summary, the interaction of O157 with epiphytes and the enhanced survival of immigrant *Salmonella* on plants containing epiphytic bacteria supports the proposition that resident plant microflora are critical for the establishment and survival of human pathogens in vegetables and may be considered a risk factor for foodborne infections.

### Bacterial traits associated with plant colonization

Enteric bacteria exhibit different levels of plant colonization within members of the same species. For example, *E. coli* K-12 colonized the lettuce rhizoplane at higher cell densities than serotype O157:H7 (49). The colonization of lettuce cultivars by *S. enterica* was also shown to be significantly influenced by serovars (57). Nevertheless, the genetic components responsible for differences in the colonization levels of these bacteria have yet to be determined. Recent studies have focused on investigating the relationships between the phenotypic and phylogenetic prevalence of traits and the ability of enteric bacteria to colonize leafy green vegetables (67, 75). These studies have identified phenotypes and genetic mutations in bacteria that are linked to the increased survival and colonization of leafy greens. Méric *et al.* (67) described the phylogenetic distribution of traits associated with plant colonization by *E. coli*. Plant-associated *E. coli* had a greater ability to produce extracellular matrix and form biofilms than a group of strains from human and other mammalian hosts. Moreover, significant differences were observed in the utilization of common carbon sources between strains of *E. coli* isolated from spinach and rocket salad and their mammalian counterparts. Isolates from plants reached lower growth yields on many carbon sources used by mammalian *E. coli*, but could utilize abundant plant compounds such as sucrose, raffinose, and *p*-hydrophenylacetic acid at higher frequencies (67). Phlyogenetic analyses revealed that although vegetable-associated *E. coli* strains were distributed amongst all major phylogroups (A, B1, B2, D, and E), the majority of the plant isolates clustered with the B1 phylogroup. In contrast, *E. coli* strains of animal origin clustered mainly with phylogroups A and B2 (67). These findings indicated that plant-associated *E. coli* were phenotypically and phylogenetically distinct from strains found in humans and mammals. Strains associated with plants in particular have been shown to harbor specific traits that facilitate their adaptation to non-host environments. Such traits include the effective utilization of carbon sources abundant in plant tissues, the production of extracellular matrix, and biofilm formation. Selective pressures unique to the plant surroundings may affect bacterial phylogroups and consequently select variable *E. coli* populations in the vegetable environment. Therefore, *E. coli* strains will differ in their ability to occupy various ecological niches and may also have different transmission ecologies throughout the food chain (67).

In an effort to identify the genetic traits associated with *E. coli* strains commonly involved in foodborne disease, Parker *et al.* (75) compared the expression profiles of *E. coli* isolates temporarily or geographically associated with the 2006 bagged-spinach outbreak. The bacteria analyzed included strains obtained from water, soil, animal feces, spinach bags, and clinical samples collected from outbreak victims. The findings obtained revealed that *E. coli* strains isolated from spinach bags and clinical samples expressed lower levels of *rpoS*-regulated genes than their environmental counterparts. Accordingly, these strains exhibited different types of mutations in the *rpoS* gene and were sensitive to oxidative, osmotic, and acid stresses. Nutrient scavenging was more apparent in the *rpoS* mutants isolated from spinach bags than in other *E. coli* strains. These findings were in accordance with the stress protection and nutritional competence hypothesis (SPANC), which states that certain environments select for *rpoS* mutants in *E. coli*, generating strains with lower tolerance to stresses, but higher nutrient scavenging capabilities. Increased nutrient scavenging allows *E. coli* to survive and adapt to new environments. Hence, it is possible that mutations in *rpoS* gene were selected when O157:H7 strains were exposed to the bagged spinach environment, and the increased nutrient scavenging ability of these cells allowed them to survive outside their normal host and infect humans (75). These findings suggest that the passage of *E. coli* strains from “field to fork” leads to mutations that are important for the survival and persistence of this pathogen during contamination and infection cycles.

## Molecular interactions mediating attachment and persistence of enteric pathogens in leafy greens

### Genes mediating the molecular interactions of *Enterobacteria* with leafy greens

Functional genomics studies have provided valuable information regarding the molecular mechanisms mediating the interactions of enteric pathogens with leafy greens. Most genes involved in the molecular interactions of human pathogens with vegetables can be grouped in four categories: those involved in cell surface structures, virulence, motility, and biofilm formation. Early reports described the relevance of genes encoding virulence factors and cell surface structures in the microbial attachment and colonization of vegetable tissues. Carey *et al.* (19) tracked the expression of a set of virulence genes in *E. coli* O157:H7 on lettuce leaves using quantitative reverse transcriptase polymerase chain reaction (qRT-PCR). Some virulence genes, such as *intimin* and *stx* genes, were induced on lettuce leaves (19) and leaf lysates (62).

In *E. coli* strains O157:H7 and K-12 and *S. enterica*, genes encoding aggregative fimbriae/curli (*agf* in *Salmonella* and *csg* in *E. coli*) have been shown to mediate the binding of these organisms to lettuce leaves and alfalfa seedlings (55, 64). The production of cellulose and the *O*-antigen capsule was also shown to be relevant for the attachment of *Salmonella* strains to the surface of leafy greens. The reduced attachment and colonization of alfalfa sprouts were reported among *Salmonella* Newport and Enteritidis mutants deficient in bacterial cellulose synthesis (*bcsA*) and *O*-antigen capsule assembly and translocation (*yihO*) (3, 4). In *E. coli* O157:H7, the attachment capacity to alfalfa sprouts was significantly lower in mutants deficient in cellulose production than in the wild type, which suggested the importance of cellulose in plant colonization (66). In contrast, Macarisin *et al.* (64) reported that while curli production was essential for the strong attachment of *E. coli* O157:H7 to spinach leaves, cellulose production was dispensable (64). This finding is in agreement with a study by Uhlich *et al.* (91), which showed that some strains of *E. coli* O157:H7 did not produce cellulose as part of their extracellular matrix.

Curli/fimbriae, cellulose, and the *O*-antigen capsule constitute the extracellular matrix, a polymeric structure important for attachment to surfaces, biofilm formation, multicellular behavior, and stress tolerance (3). These structures promote bacterial attachment to animal cells and play a critical role in biofilm formation and host colonization. Plant-symbiotic and pathogenic bacteria utilize curli/fimbriae and cellulose to anchor to plant surfaces (90). Curli and cellulose have been shown to be important for the attachment of enterobacteria to leafy greens, which indicates that these organisms behave similarly to plant-associated bacteria during the colonization of plant tissues. Therefore, cellular structures used by human pathogens to invade animal hosts may also be important for their interaction and establishment in vegetables tissues.

Motility and chemotaxis genes are required for the early stages of the interaction of enteric pathogens with vegetables. *Salmonella* exhibited chemotactic responses towards leaf exudates and required flagellar genes for efficient attachment and internalization into lettuce leaves (59). Similarly, mutations in *fliC*, the gene encoding flagellin, resulted in the reduced attachment of the *S. enterica* serovar Senftenberg to basil leaves (6). In contrast, the same mutation in the *S. enterica* serovar Typhimurium did not lead to any attachment defects (6). The deletion of *fliN*, a gene involved in flagellum biosynthesis, in *E. coli* strain K-12 produced cells with a reduced capacity to attach to the rhizoplane (50), and this phenotype was not observed in *E. coli* O157:H7 associated with lettuce roots or leaves. These findings indicated that although motility and chemotaxis were important for the microbial colonization of vegetables, their effect may vary depending on bacterial genotypes and environmental conditions.

Proteins involved in biofilm formation also facilitate the establishment of enteric pathogens in leafy greens. The *ycfR* gene, which is involved in tolerance to multiple stresses and the development of biofilms, was previously shown to be critical for the attachment and persistence of *E. coli* and *S. enterica* on vegetable tissues. In *E. coli* strains K-12 and O157:H7, *ycfR* mutants were impaired in the long-term colonization of leaf surfaces (38). Deng *et al.* (28) also showed that the survival of *ycfR* deletion mutants in *E. coli* O157:H7 Sakai was reduced on spinach leaves treated with sub-lethal concentrations of chlorine. In the *S. enterica* serovars Typhimurium and Saintpaul, *ycfR* was shown to be important for their attachment to intact spinach leaves and grape tomatoes. The deletion of *sirA*, a global regulator of biofilm formation in *Salmonella* species, also produced cells with impaired surface attachment and long-term survival on spinach leaves (81).

A recent study by Fink *et al.* (38) demonstrated that, in addition to *ycfR*, a gene that represses biofilm formation, *ybiM*, was upregulated in both *E. coli* K-12 and O157:H7 associated with lettuce leaves. *ybiM* mutants were slower to attach to lettuce leaves, but eventually reached levels of colonization similar to the wild type. Collectively, these studies illustrate that genes important for biofilm formation also have a role in the mechanisms used by enterobacteria to colonize the surface of fresh vegetables. Whether attachment and persistence on leafy greens are processes mediated by the formation of biofilm structures or by different mechanisms employing similar genetic responses are questions that have yet to be answered.

### Global gene expression changes in responses to interactions with the phyllosphere and rhizosphere of leafy greens

A key step in elucidating the mechanisms that allow enteric pathogens to survive and colonize leafy greens is to understand the global transcriptional responses triggered by the association of these organisms with plant tissues. Recent studies have focused on transcriptomic analyses of common foodborne pathogens, specifically *E. coli* and *Salmonella* strains, existing in close contact with the phyllosphere of lettuce and spinach plants. This research has shown that foodborne pathogens exhibit distinct changes in gene expression upon association with the phyllosphere of intact and damaged fresh produce.

Fink *et al.* (38) investigated the transcriptional responses of the *Escherichia coli* strains K-12 and O157:H7 associated with intact lettuce leaves. The global transcriptome of these bacteria when they associated with the lettuce phyllosphere was characterized by stress responses triggered by nutrient limitation. Upregulation of genes involved in cell envelope stress, the sulfur and phosphate starvation regulons, and the *rpoS* regulatory network illustrated these responses. Interactions with the leaf surface led to the downregulation of genes encoding proteins involved in energy metabolism and transport processes. In contrast, genes regulating the formation of biofilms and curli fibers were expressed at high levels under these conditions. The induction of the curlin subunit genes, *csgA* and *csgB*, as well as the biofilm regulators, *ycfR* and *ybiM*, was more than tenfold in cells associated with lettuce leaves. Attachment and colonization experiments revealed that the ability of *csgA* and *ycfR* mutants in *E. coli* K-12 and O157:H7 to attach to lettuce leaves was reduced, and these mutants were impaired in the long-term colonization of the leaf surface. These findings were consistent with previous studies that demonstrated the relevance of cell surface structures and biofilm formation in the establishment and colonization of plants by enteric pathogens.

A time-course experiment comparing the transcriptional responses of phyllosphere associated K-12 and O157:H7 over three days showed that adaptation to the leaf environment was characterized by an overall decrease in the expression of genes mediating cellular energy and metabolism, especially those involved in the synthesis of ribosomal RNA and iron homeostasis (38). A transient increase was also observed in the expression of genes involved in energy homeostasis, particularly those mediating the synthesis of deoxyribonucleotides from ribonucleotide precursors, but most genes returned to their normal expression levels by day three. These findings are characteristic of a classic response to nutrient limitation and support the hypothesis that enteric pathogens survive and propagate in leafy greens by inducing physiological responses that allow them to cope with the scarcity of food sources encountered on vegetable surfaces.

Enteric pathogens can establish niches on fresh produce by taking advantage of the substances released by damaged plant tissues during the harvesting and processing of vegetables. Kyle *et al.* (62) used lettuce lysates as model systems to mimic the chemical conditions found at wound sites in processed lettuce. The findings of this study showed that *E. coli* O157:H7 grew and quickly multiplied in lettuce lysates after a short adaptation period of four to five hours. A transcriptional profile analysis of this bacterium after exposure to lettuce lysates revealed several adaptations that may allow the survival and persistence of this organism in a non-host environment, such as a vegetable processing facility. The expression of genes encoding proteins involved in carbohydrate utilization and transport was increased in O157:H7 cells exposed to lettuce lysates. These proteins may have allowed pathogenic *E. coli* to use the sugar substrates released by injured lettuce leaves as alternative carbon sources to grow and propagate outside their normal host (62).

The exposure of pathogenic *E. coli* to lettuce lysates revealed the strong induction of genes involved in oxidative stress and DNA repair. Lettuce lysates were shown to contain high levels of hydrogen peroxide and other reactive oxygen species (62), which suggested that bacterial cells may use an increased oxidative stress response as a mechanism to cope with the high concentrations of oxygen radicals encountered early during their exposure to injured leaf tissues. A decrease in the concentrations of reactive oxygen species was shown to create a suitable environment for the subsequent growth and persistence of O157:H7 in damaged plant tissues several hours after these cells associated with lysates.

The strong oxidative stress response elicited by pathogenic *E. coli* upon its exposure to lettuce lysates, may have allowed this organism to survive after treatments with oxidative sanitizers such as calcium hypochlorite and hydrogen peroxide (62). Initial contact with injured plant tissues prepares this organism to cope with the scarce nutrient conditions and sanitation practices encountered in the vegetable-processing environment. These findings help explain the common association of *E. coli* O157:H7 with foodborne illnesses linked to the consumption of fresh produce.

While several research groups have studied the transcriptional responses of *E. coli* strains associated with the phyllospheres of different vegetables; fewer studies have investigated changes in the gene expression of *S. enterica* growing on lettuce leaves. Kroupitski *et al.* (60) examined the genes involved in the persistence of *S. enterica* on post-harvested lettuce during cold storage using recombinase-based *in vivo* expression technology, RIVET (60), and identified thirty-seven genetic loci that were induced upon the association with lettuce leaves under conditions mimicking post-harvest storage. These genes encoded proteins involved in stress responses, pathogenicity, metabolism, and regulatory functions. Further analyses showed that the survival of knockout mutants in four of the over-expressed genes, *stfC*, *bcsA*, *misL*, and *yidR*, was decreased on intact lettuce leaves after seven days of cold storage. The proteins encoded by these genes included a fimbrial outer membrane usher protein (*stfC*), an adhesin expressed from a *Salmonella* pathogenicity island (*misL*), a cellulose synthase subunit (*bcsA*), and a putative ATP/GTP binding protein (*yidR*). In spite of the decreased survival of the *stfC* mutants on lettuce leaves, these cells displayed attachment and biofilm formation phenotypes similar to those of the wild type strain. In contrast, *misL*, *bcsA* and *yidR* were defective at surface attachment on lettuce leaves and had reduced biofilm formation phenotypes. These results are consistent with the physiological roles of *misL*, *bcsA*, and *yidR*, which have previously been shown to mediate the attachment of *Salmonella* to various surfaces. The findings of these studies indicated that the genes involved in surface attachment and biofilm formation in *S. enterica* are important for survival on and colonization of the lettuce phyllosphere.

*misL* encodes an intestinal colonization factor that binds fibronectin, and is essential for the persistence of *S. enterica* in a mouse infection model (30). These findings are consistent with a study that investigated the transcriptome of *S. enterica* in the phyllosphere of soft-rotted lettuce and cilantro compared to growth on LB broth (46). The results of the experiment revealed that *misL* and other genes important for the colonization of *Salmonella* primary mammalian hosts were overexpressed when associated with macerated lettuce and cilantro leaves. These findings suggest that the high level of adaptation to and persistence of enteric pathogens in leafy greens may be due to a niche overlap between their primary mammalian hosts and the conditions present in damaged vegetable tissues during post-harvest conditions.

The global gene expression profiles of *E. coli* strains K-12 and O157:H7 associated with lettuce roots were recently examined by Hou *et al.* (49, 50). These studies revealed that the expression of genes involved in energy metabolism, cellular processing, and biofilm formation were higher in both organisms when they lived in the lettuce rhizosphere.

Marked differences were observed in the expression of genes involved in protein synthesis among *E. coli* strains. These genes in *E. coli* K-12 were mostly upregulated upon colonization of the root system (50). In contrast, O157:H7 protein synthesis-related genes were repressed in cells growing on the rhizoplane (49). The increased expression of protein synthesis genes in the K-12 strain indicates that this organism can easily adapt to and survive in the root environment. The large number of protein synthesis genes repressed by *E. coli* O157:H7 suggests poor adaptation to the rhizosphere and may also explain the low levels of root colonization observed in this organism. These findings are in accordance with previously reported data describing colonization patterns of rhizoplane associated O157:H7 and non-pathogenic *E. coli* (49, 50, 93).

In spite of the close phylogenetic relationship between *E. coli* strains O157:H7 and K-12, these organisms employ different strategies to survive and colonize lettuce roots. Transcriptional analyses combined with colonization assays and confocal microscopy revealed that genes encoding a curli regulator (*ctl*), curli subunits (*csg*A) and flagella (*fli*N) were necessary in *E. coli* K-12 for its attachment to and colonization of roots (49, 50). These genes were not differentially expressed in O157:H7 cells living in association with root tissues. The stress response regulator *ycfR* was the only gene found to be essential for the attachment of pathogenic *E. coli* and its colonization of the lettuce rhizoplane.

The expression of the biofilm modulator *ybiM* was found to be increased in both *E. coli* K-12 and O157:H7. However, *ybiM* mutants attached to and colonized lettuce roots at levels similar those of the wild type. These findings suggest that the mechanisms for attachment and adaptation of pathogens to the rhizosphere of vegetables may be strain-specific. Therefore, caution must be used when developing intervention strategies to control the contamination of leafy greens by enteric pathogens since strains of the same bacterial species might respond differently to sanitation treatments.

## Model for the association and establishment of enteric pathogens with leafy green vegetables

**Fig. 2:**
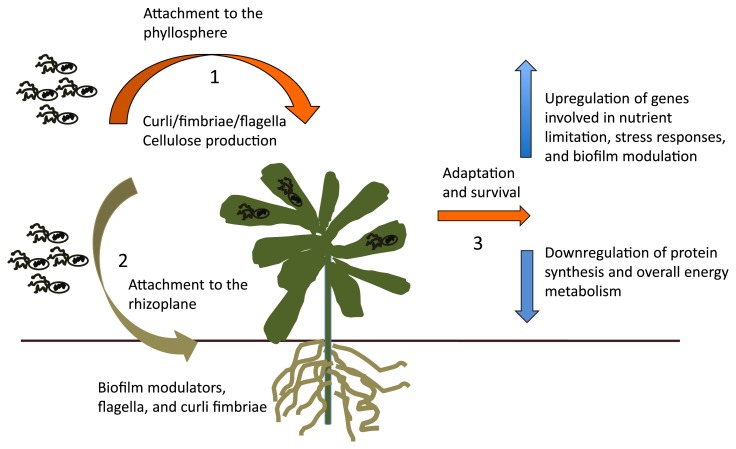
Molecular mechanisms mediating the colonization of leafy greens by human pathogens. Schematic diagram illustrating the most relevant aspects of molecular processes that facilitate the association and colonization of leafy green vegetables by enteric bacterial pathogens. The initial attachment to the plant rhizosphere and phyllosphere is facilitated by cell surface structures important for surface attachment and biofilm formation (steps 1 & 2). Survival in the harsh vegetable environment is mediated by the induction of stress responses and downregulation of genes involved in protein synthesis, energy, and metabolism (step 3).

Research on the molecular interactions of enteric bacteria with plants has shed light on the strategies that human pathogens employ to survive and persist in leafy green vegetables. The availability of genomic techniques has allowed the identification of genes, proteins, and molecular responses necessary for effective colonization and survival in plant tissues. We constructed a model based on transcriptomic data and mutational studies to explain and visualize important molecular events mediating the association of human pathogens with leafy green vegetables (Fig. 2).

Initial colonization involves attachment to plant tissues and is driven by cellular structures, specifically curli/fimbriae and flagella. This proposition is supported by several studies linking differential binding to plant structures to the ability of various strains of *E. coli* and *S. enterica* to produce curli and express flagellar genes (6, 50, 63, 81, 100). In spite of evidence to support the role of cell surface structures in mediating attachment to leafy greens, it is important to consider the marked levels of variations in attachment phenotypes among pathogenic bacteria of the same species. In some cases, isogenic mutants of the genes encoding curli subunits and flagella proteins do not exhibit significant attachment defects (38, 49, 50). Thus, these proteins may play a role in one or more of the pathways mediating attachment to the surface of leafy greens. These processes are governed by complex molecular connections that may involve one or multiple mechanisms depending on the plant and bacterial genotypes present in a given environment.

Current research strongly suggests that genes encoding proteins involved in biofilm formation are important for the attachment and persistence of enteric bacteria in the phyllosphere and rhizosphere of leafy greens (Table 2). The ability to form biofilms under laboratory conditions has been associated with enhanced plant colonization in various strains of *E. coli* and *S. enterica* (61, 67, 76, 77). These organisms have been shown to form biofilms under conditions mimicking the food-processing environment (20, 71, 91). However, the occurrence of mature biofilms formed by enteric pathogens in natural plant environments has not yet been documented. Therefore, whether the formation of mature biofilms is a factor that is important for the colonization of leafy greens by pathogenic bacteria remains to be elucidated.

Studies by Fink *et al.* (38) and Hou *et al.* (49) showed that *ybiM*, a gene that triggers the inhibition of biofilm formation by overproducing colanic acid, was overexpressed in *E. coli* strains associated with the rhizoplane and phyllosphere of lettuce plants. These findings suggested that the suppression of biofilm formation was part of the adaptation process necessary for the colonization of plant tissues. Proteins involved in biofilm formation may facilitate the association of human pathogens with naturally occurring plant-specific biofilms. This hypothesis is supported by several studies that have reported the occurrence of native biofilms formed by indigenous microbial flora on the surface of alfalfa sprouts and other vegetables (36, 37). These naturally occurring biofilms have been proposed to provide protective colonization sites for enteric pathogens such as *E. coli* O157:H7 and *Salmonella* (36). Another possibility is that the genes involved in biofilm modulation are part of the stress responses necessary for survival in the plant environment and fulfill physiological functions different from the formation of mature biofilms. In summary, components of the biofilm formation machinery are important components for the interaction of enteric bacteria with fresh produce; however, the detailed molecular mechanisms by which this occurs have yet to be elucidated.

Expression profiling data on enteric microbe-plant interactions indicate that the association with leafy greens triggers the differential expression of many genes involved in survival and adaptation to harsh conditions. Therefore, one of the crucial steps necessary for colonization and persistence in hostile plant environments is the induction of strong stress responses. Some of the stress responses characteristic of adaptations to the plant environment involve the oxidative (*oxyR*), cell envelope (*pspABC*), and nutrient limitation (*rpoS*) regulons (38, 62, 75). These mechanisms allow human pathogens to cope with scarce nutrients, reactive oxygen species, antimicrobial substances, and defense responses, all of which threaten survival in the plant environment (38, 62, 75). Following the induction of stress responses, enteric bacteria adjust their metabolism to conserve energy and scavenge nutrients efficiently. Transcriptional analyses strongly suggest that a downshift in bacterial metabolism is triggered upon colonization of the phyllosphere and rhizoplane of leafy greens. This adaptation is illustrated by the reduced expression of genes involved in protein synthesis and utilization of common carbon sources. In contrast, survival and persistence on or within leafy greens is achieved by increasing the expression of genes necessary for the consumption of carbohydrates and substrates unique to plant tissues.

## Molecular interactions of human pathogens on leafy greens: implications for food safety

Studies on the molecular events triggered by the association of enteric pathogens with leafy green vegetables have improved our understanding of the mechanisms by which these organisms survive outside their normal host. One of the most relevant findings from transcriptional analyses and mutational studies was that enteric bacteria may colonize vegetables using the same cellular structures that mediate attachment to animal cells (9, 55, 63, 64, 82). Moreover, exposure to the vegetable environment stimulates cellular responses that overlap those required to colonize the animal host intestine (46). These findings raise the question of whether survival on vegetable tissues alters the physiology of enteric pathogens, thereby enhancing their ability to infect their normal host. The level of enteric bacteria cells on leafy-green crops may not be the only determinant linked to their ability to cause foodborne outbreaks. Alternatively, physiological changes triggered by survival in the plant environment may allow enteric bacteria to more effectively cause disease in humans and at lower infectious doses.

Another finding obtained from functional genomics studies is that enteric pathogens can grow on damage vegetable tissues (11, 62). In addition, *E. coli* O157:H7, upon exposure to injured leaves, has been shown to activate transcriptional responses that enhance its resistance to oxidative agents (62). The fresh produce industry uses oxidative compounds, such as hydrogen peroxide and sodium hypochlorite, to wash and sanitize vegetables during post-harvesting processing. Enteric pathogens show increased resistance to these disinfectants after exposure to wounded plant tissue, which implies that survival in the vegetable environment allows them to cope with decontamination treatments and persist in fresh produce. The high level of tolerance to disinfectants together with their ability to grow in damage-plant tissue may explain the common association of enteric pathogens with foodborne outbreaks in leafy green vegetables.

Plant cultivars have been shown to influence the metabolic activity and colonization capabilities of enteric bacteria associated with leafy greens (57, 79). Similarly, specific bacterial traits have recently been associated with the ability of human pathogens to colonize and persist in plants (57, 67). These findings suggest that breeding pathogen-resistant crops may be a way to ameliorate the problem of foodborne outbreaks associated with the consumption of vegetables. In contrast, the identification of genetic determinants specific to enteric bacteria associated with plants may lead to the development of markers to monitor crops and production facilities for the presence of human pathogens with strong plant colonization potential.

The ability to produce biofilms as well as the differential expression of genes involved in biofilm modulation, are characteristics linked to the association between and colonization potential of certain human pathogens on leafy greens. Moreover, biofilm formation on the surface of lettuce and spinach has been shown to reduce the efficacy of sanitation treatments in food processing plants (71). It is tempting to speculate that future food safety efforts should target the inhibition of biofilm formation pathways through quorum sensing inhibitors or compounds that block the synthesis of essential bacterial biofilm components, such as curli and cellulose. Recent studies have suggested that selected essential oils and competition with probiotic bacteria may be effective treatments to inhibit biofilm formation by foodborne pathogens (56, 98). These strategies may also be applied to development interventions for reducing the biofilm-formation capabilities of some foodborne pathogens.

Enteric foodborne pathogens can enter the VBNC state while interacting with foods at different stages of the food production chain (2, 29, 31, 65). This dormant state prevents the effective detection of microbial populations in fresh produce. This is a highly relevant issue given the potentially low infectious dose for human enteropathogens, such as *E. coli* O157:H7. Although methods to identify cells in the VBNC state have only been developed for scientific research, novel molecular technologies present an opportunity to further enhance the applicability, accuracy, and accessibility of these techniques. While the transmission of pathogens in the VBNC state through foods has been suggested, very few studies have addressed this issue. Therefore, the VBNC state remains an area of food safety research that should be further explored. Detailed knowledge of the magnitude of the VBNC state and of the food safety risk associated with the non-culturable response, in addition to improved detection methods, is of critical importance for developing effective intervention strategies and ameliorating leafy-green contamination by human pathogens.

## Figures and Tables

**Table 1:** Prominent foodborne outbreaks associated with the consumption of leafy greens and sprouts contaminated with enteric pathogens between 2006 and 2012. The data presented in the table was obtained from current literature reviews and the Centers of Disease Control and Prevention (2013).

Bacterial strain	Produce type	Year	Number of cases	Reference	Country
*Escherichia coli* O157:H7	Spinach	2012	33	http://www.cdc.gov/ecoli/2012/O157H7-11-12/	USA
*E. coli* O26	Clover sprouts	2012	29	http://www.cdc.gov/ecoli/2012/O26-02-12/	USA
*E. coli* O157:H7	Romaine Lettuce	2011	60	(84)	USA
*E. coli* O104	Fenugreek seeds	2011	4,075	(18, 22, 45)	Germany, France, USA
*Salmonella enterica* sv. Enteritidis	Alfalfa sprouts	2011	25	http://www.cdc.gov/salmonella/sproutsenteritidis0611/070611/index.html	USA
*E. coli* O145	Lettuce	2010	27	(89)	USA
*S. enterica* sv. Newport	Alfalfa sprouts	2010	44	http://www.cdc.gov/salmonella/newport	USA
*S. enterica* sv. SaintPaul	Alfalfa sprouts	2009	228	(23)	USA
*S. enterica* sv. Bovismorbificas	Alfalfa seeds	2009	42	(80)	Finland
*E. coli* O157:H7	Lettuce	2007	50	(42)	Iceland, the Netherlands
*S. enterica* sv. Stanley	Alfalfa sprouts	2007	44	(97)	Sweden
*S. enterica* sv. Weltevreden	Alfalfa sprouts	2007	45	(32)	Norway, Denmark, Finland
*E. coli* O157:H7	Spinach	2006	205	(21)	USA
*E. coli* O157:H7	Lettuce	2005	135	(85)	Sweden

**Table 2:** Genes involved in the association of *Enterobacteria* with leafy green vegetables.

Genes	Function	Organism	References
*csgA*	Curli formation; curlin major subunit	*E. coli* K-12	(38, 50)
*crl*	Regulation of curli formation	*E. coli* K-12	(38, 50)
*fliC*	Biosynthesis of flagella	*Salmonella enterica* sv. Senftenberg	(6)
*fliN*	Biosynthesis of flagella	*E. coli* K-12	(50)
*espA*	Protein translocator, TTSS encoded by the LEE island	*E. coli* O157:H7	(82)
*ycfR*	Stress response regulator, biofilm modulation	*S. enterica* sv. Typhimurium *E. coli* O157:H7, *E. coli* K-12	(38, 50, 81)
*yidR*	Putative ATP/GTP binding protein	*S. enterica* sv. Typhimurium	(60)
*bcsA*	Biosynthesis of cellulose	*S. enterica* sv. Typhimurium	(60)
*agfAB*	Regulation and assembly of aggregative fimbriae	*S. enterica* sv. Enteritidis	(3, 4)
*misL*	Adhesin expressed from pathogenicity island-3	*S. enterica* sv. Typhimurium	(60)
*yigG*	Putative inner membrane protein of unknown function	*S. enterica* sv. Typhimurium *S. enterica* sv. Saintpaul	(81)
*ybiM*	Regulator of biofilm formation through the production of colanic acid	*E. coli* O157:H7, *E. coli* K-12	(38)
*sirA*	Response regulator involved in biofilm formation	*S. enterica* sv. Saintpaul *S. enterica* sv. Typhimurium	(81)
